# Case Report: Endoscopic anal papillectomy combined with ligation: a new approach for the treatment of grade IV hemorrhoids (with video)

**DOI:** 10.3389/fgstr.2026.1681972

**Published:** 2026-04-07

**Authors:** Xiaopan Lv, Mingli Zhu, Xianzhong Zeng, Zheng Li, Kaitong Jiang, Xuemin Yuan

**Affiliations:** 1 School of Clinical Medicine, Shandong Second Medical University, Weifang, Shandong, China; 2 Department of Gastroenterology, Weihai Central Hospital, Weihai, Shandong, China; 3 Department of Gastroenterology, The People’s Hospital of Linyi, Linyi, Shandong, China; 4 Department of Gastroenterology, Jinluo Hospital, Linyi, Shandong, China

**Keywords:** endoscopic anal papillectomy, endoscopic ligation, grade IV hemorrhoids, new approach, snare, treatment

## Abstract

**Background and aims:**

Hemorrhoids represent the most prevalent anorectal disorder globally. They are categorized into three types based on location: internal, external, and mixed hemorrhoids. Furthermore, internal hemorrhoids are classified from I to IV, depending on their appearance and degree of prolapse. Surgical intervention is frequently required for Grade III-IV and mixed hemorrhoids, which exhibit complex and severe symptoms. The challenges of these surgical procedures include extensive incisions, substantial intraoperative bleeding, intense postoperative pain, and a heightened risk of complications. Consequently, the exploration of alternative therapies continues.

**Methods:**

This report introduces an innovative endoscopic technique employed to treat a female patient suffering from Grade IV hemorrhoids for three years. Utilizing an endoscope, the prolapsed hemorrhoid and hypertrophic anal papillae were progressively excised with a snare, followed by the application of a ligator to ligate the lax mucosa above the dentate line at multiple sites, thereby preventing future prolapse.

**Results:**

The procedure resulted in minimal bleeding, reduced postoperative pain, a shorter recovery period, and the patient experienced no complications such as anal stenosis or recurrent prolapse during the 8-month follow-up.

**Conclusion:**

This technique is particularly innovative as endoscope facilitates a clearer surgical view and more precise manipulation, thus minimizing damage to the rectal mucosa and anal skin. Furthermore, this technique can be utilized to concurrently remove hemorrhoids during gastrointestinal endoscopy, which will alleviate the patient’s multiple suffering and save money and time. This method offers a new direction for treating Grade IV hemorrhoids. Longer follow-up periods and larger sample size comparative studies are needed in the future to verify its safety and efficacy.

## 1 Introduction

Hemorrhoids are the most prevalent anorectal disease globally. An early survey conducted in the United States by Johanson and Sonnenberg in 1990 reported a hemorrhoid prevalence of 4.4% (1). Studies from Austria and South Korea indicate adult prevalence rates of 38.9% and 14.4%, respectively (2, 3). It is estimated that nearly half of the population over 50 years old will experience symptoms at least once (4).

Hemorrhoids are categorized by location into three types: internal (originating above the dentate line), external (below the dentate line), and mixed type (5). Internal hemorrhoids are further classified into four grades based on appearance and prolapse severity. Grade I hemorrhoids do not prolapse; Grade II prolapse but spontaneously reduce; Grade III require manual reduction; and Grade IV are irreducible and may include acute thrombosis or strangulation (6).

Several clinic-based interventions, such as rubber band ligation, infrared coagulation, sclerotherapy, laser therapy, and radiofrequency ablation, are applicable for managing Grades I-II and some Grade III hemorrhoids (8–11). However, Grade III-IV and mixed hemorrhoids with severe symptoms typically require surgical intervention. The conventional hemorrhoidectomy, known as the Milligan-Morgan hemorrhoidectomy (MMH), remains the standard for Grades III and IV due to its low recurrence rate (7). Traditional surgical approaches, however, involve extensive incisions that often result in severe postoperative pain, prolonged recovery, and complications such as bleeding and anal stenosis (12). For Grades III-IV hemorrhoids, several new alternative therapies are in use, including stapled hemorrhoidopexy, transanal hemorrhoidal dearterialization, doppler guided hemorrhoidal arterial ligation with recto-anal-repair (13–15). These methods decrease the incidence of complications and postoperative pain (13–16). While these technologies have made progress, they still have not fully addressed the core need for real-time, high-definition, precise visualization in individualized anatomical reconstruction.

This report introduces a novel endoscopic method for treating Grade IV hemorrhoids. Utilizing an endoscope, the prolapsed hemorrhoids and hypertrophic anal papillae were progressively excised with a snare, followed by the application of a ligator to ligate the lax mucosa above the dentate line at multiple sites. It provides excellent visualization and precise manipulation, minimizes damage to the rectal mucosa and anal skin, and significantly reduces postoperative pain, allowing for a swift recovery without disrupting normal life.

## 2 Case presentation

A 58-year-old female patient presented with protruding hemorrhoidal mucosa, which had been visible for three years and was irreducible. She also experienced symptoms such as anal moisture, itching, and a sensation of obstruction. Recently, she sought inpatient treatment at our hospital due to six months of intermittent abdominal pain and persistent malformed stools without improvement. Upon examination, a large protruding hemorrhoidal mass was observed outside the anus, which was irreducible. The patient had an 8-year history of hypertension, controlled with valsartan capsules. The blood pressure was currently well controlled. A comprehensive preoperative assessment was performed, including an electrocardiogram, complete blood count, liver and kidney function tests, coagulation profile, and screening for preoperative infections, confirming her suitability for gastrointestinal endoscopic surgery. Prior to the endoscopic examination, routine intestinal cleansing was conducted.

The patient was positioned in the left lateral decubitus and underwent routine intravenous anesthesia. An endoscope was inserted up to the terminal ileum and subsequently withdrawn, revealing a 1-cm polypoid protrusion in the ascending colon. Methylene blue saline injection was performed, followed by resection using a snare. The site was closed with a titanium clip, and the specimen sent for pathological examination.

**Figure 1:**
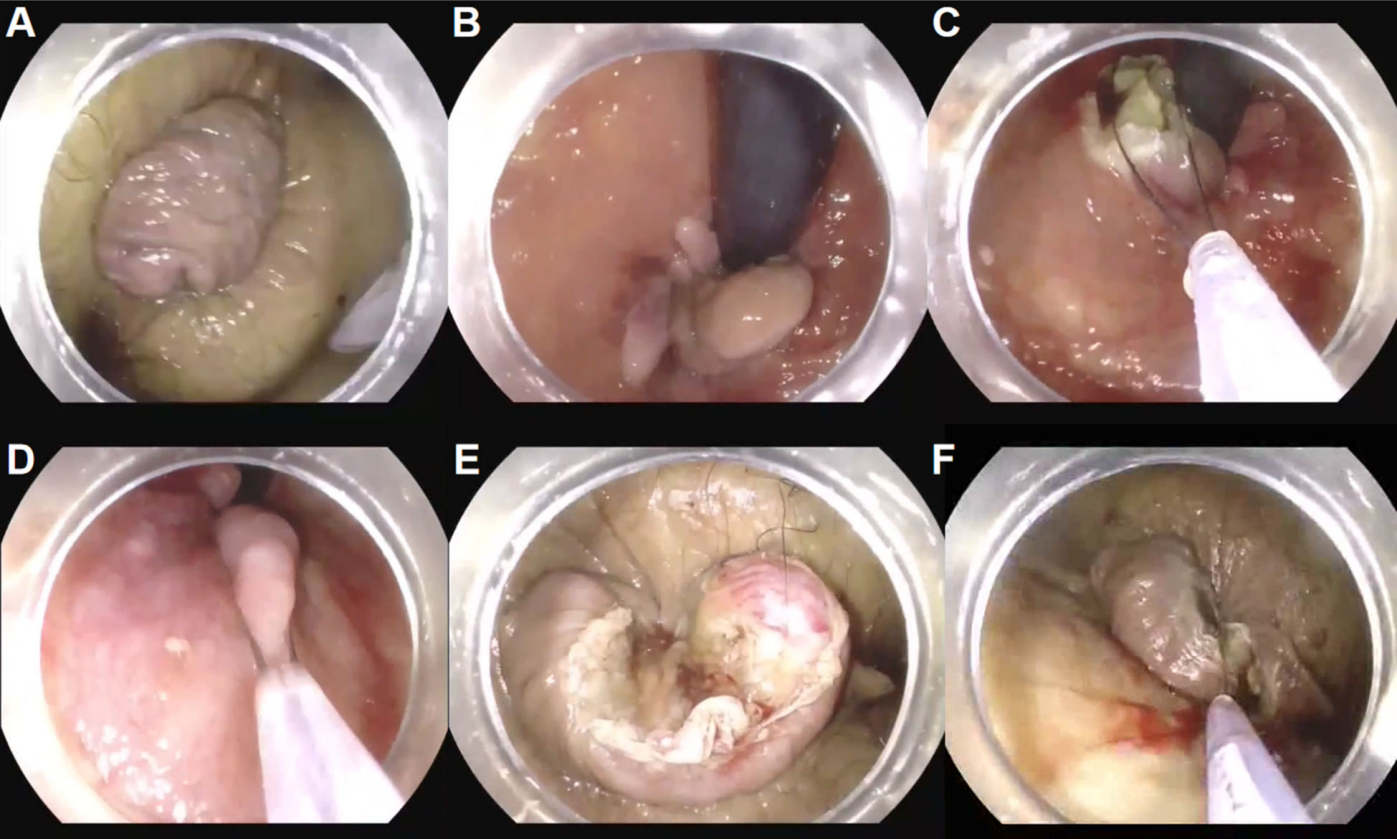
**(A)** The protruding hemorrhoidal mass outside the anus. **(B–D)** Post-reduction surgical procedure. **(E, F)** Excision of Remaining External Lesions of the Anus.

Further examination of the rectum displayed enlarged hemorrhoid and multiple hypertrophic anal papillae. Upon withdrawing the endoscope to the anal verge, the mucosa was noted to be significantly protruding and difficult to retract spontaneously (**Figure 1A**). Due to the large size of the mass impairing visualization, distinguishing between diseased and normal tissue became challenging. Initially, the mass was manually repositioned within the anal canal (**Figure 1B**). A snare was introduced through the endoscope’s working channel and positioned at the base of the mass. It was gradually tightened, and electrocautery at 40W power was used to resect part of the hemorrhoid affecting the view. Using the same method, the remaining hemorrhoid tissue and two hypertrophic anal papillae were resected (**Figures 1C, D**), with residual portions self-extruding through the anus (**Figure 1E**). The procedure resulted in minimal bleeding. The scope was then retracted outside the anus, and the remaining protruded hemorrhoidal tissue and papillae were resected in stages using a snare (**Figure 1F**). During the entire procedure, 12 anal papillae were excised. The excision sites consisted mostly of yellow-white tissue. Most of the wound surfaces did not bleed, there was localized bleeding from some areas. Hemostatic forceps were inserted through the endoscopic channel to clamp the bleeding points, and electrocautery was applied at a power setting of 30W to achieve hemostasis. Finally, a Nanjing Minimally Invasive Six-Ring Ligator was applied to the lax mucosa above the dentate line in a circumferential fashion at five primary sites to prevent further prolapse. An operation video is available for reference (Video 1).

**Figure 2:**
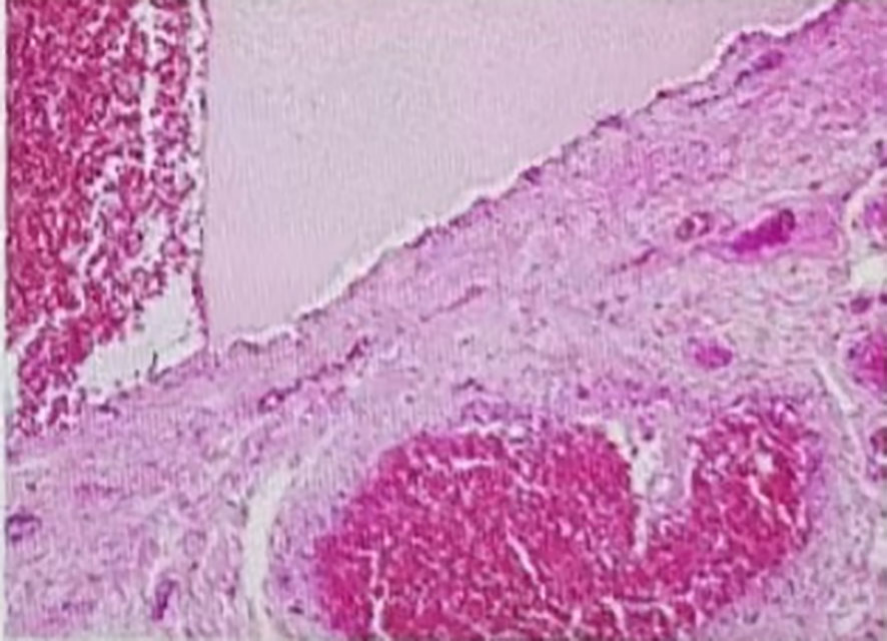
Pathology results: the mixed hemorrhoids.

**Figure 3:**
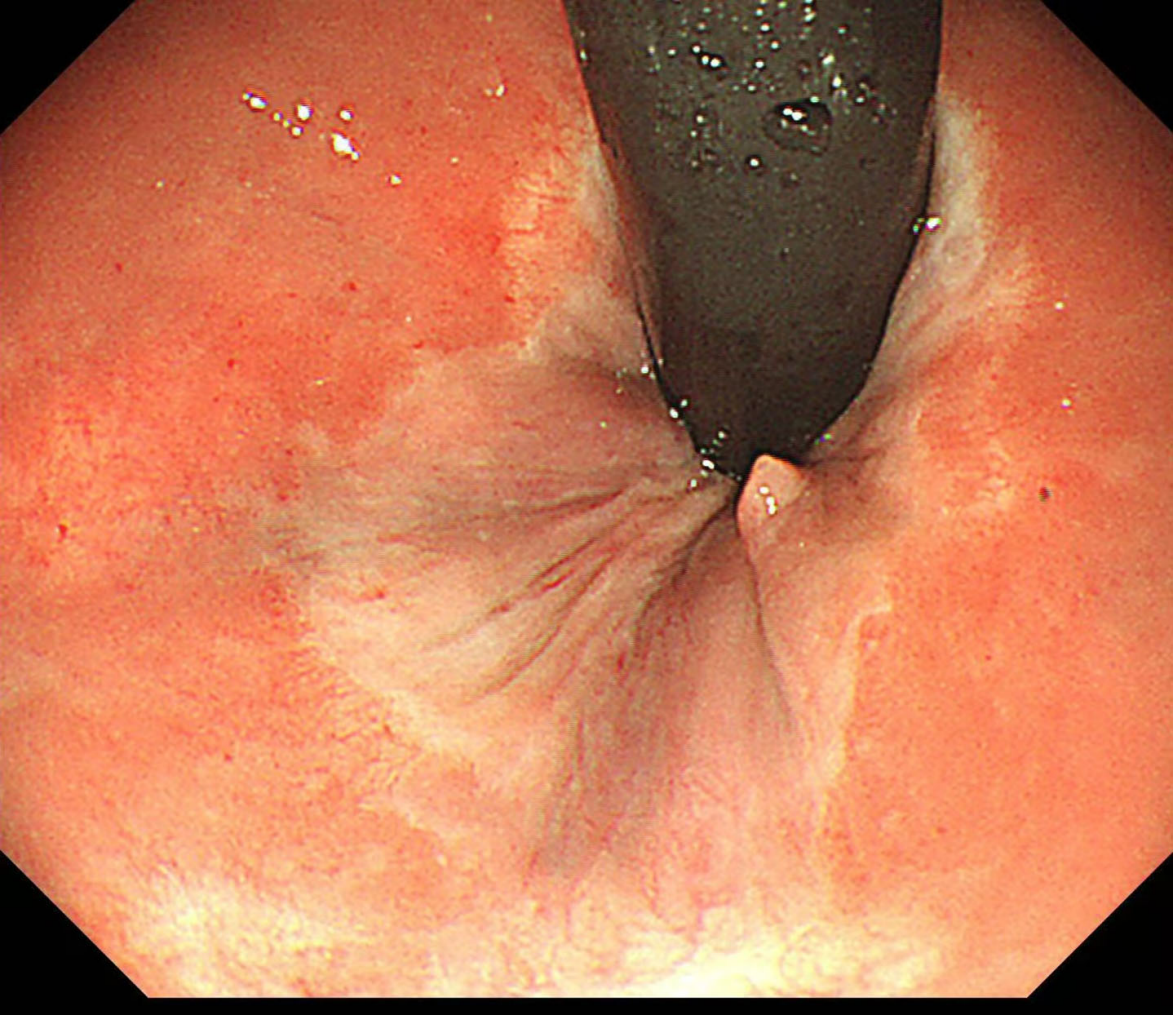
8-month postoperative endoscopic follow-up (within the anal canal).

**Figure 4:**
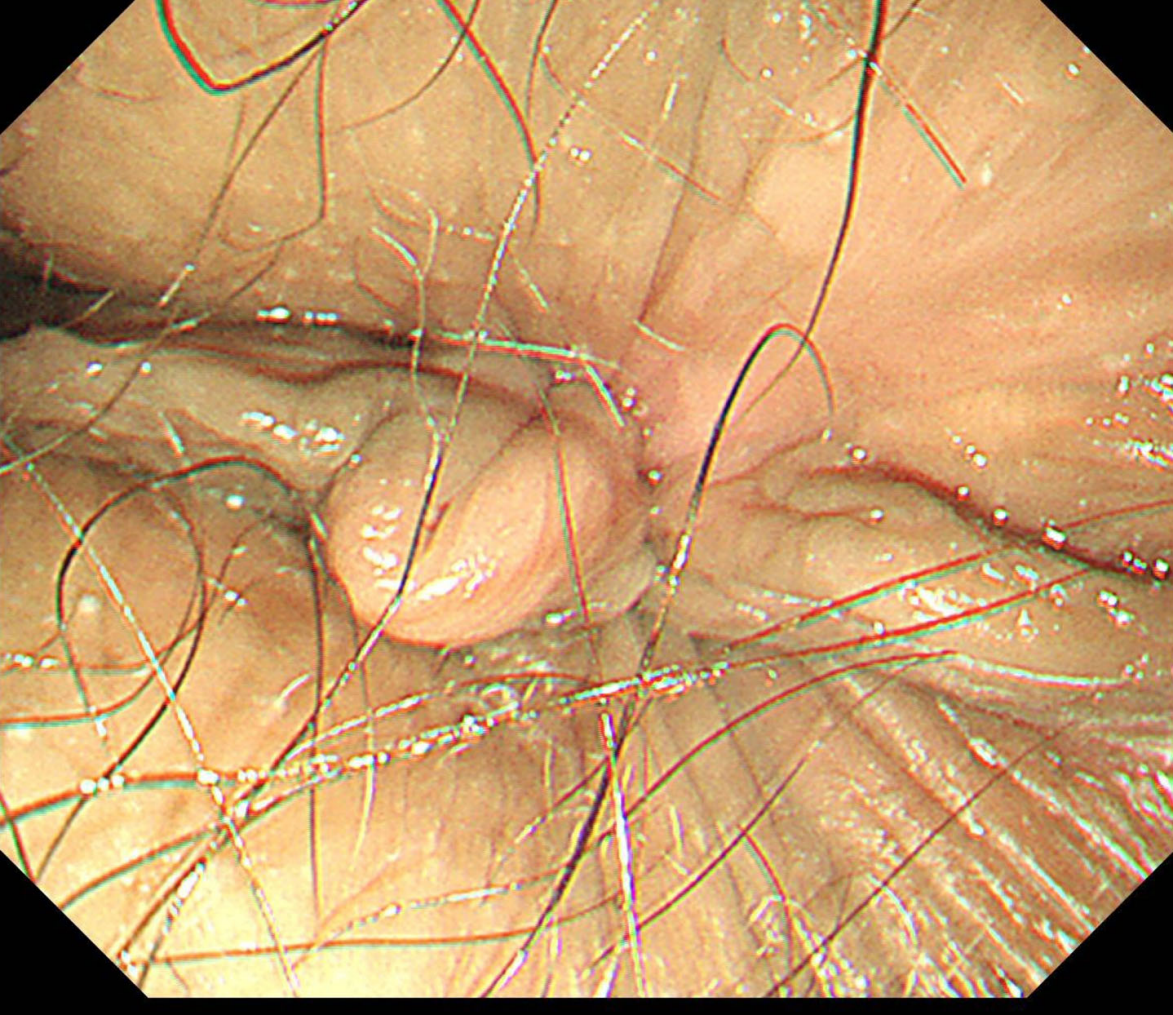
8-month postoperative endoscopic follow-up (external to the anal canal).

Postoperatively, the patient received intravenous antibiotics and Yunnan Baiyao to prevent infection and bleeding. She experienced significant pain on the night following the surgery, which was obviously alleviated with a dose of ketorolac tromethamine. Diclofenac sodium was prescribed the next day for mild pain management. The patient was able to ambulate independently shortly after the operation and was discharged six days later. Pathological examination revealed the resected anal tissue indicated mixed hemorrhoids (**Figure 2**). In the telephone follow-ups at 1 month and 6 months, the patient reported a good recovery with no abnormal bowel movements such as diarrhea, constipation or narrowing of stools, and no recurrence of bleeding or prolapse. Endoscopic follow-up at 8 months showed: No obvious scarring or stricture at the anal canal, the operative site has healed well(**Figures 3**, **4**).

## 3 Discussion

Hemorrhoids consist of congested, dilated, and tortuous blood vessels, particularly the inferior rectal veins (16). Symptoms include anal and rectal bleeding, anal pain, mucus discharge, fecal leakage, itching, and prolapse of hemorrhoidal cushions. Internal hemorrhoids primarily arise from pathological conditions such as arteriovenous fistulas and changes or displacements in vascular plexuses, while external hemorrhoids may result from thrombosis, tissue hyperplasia, blood flow obstruction, or vessel dilation beneath the mucosa distal to the dentate line (16). Grade IV Hemorrhoids often present with significant prolapse and bleeding, severely impacting quality of life.

Hypertrophied anal papillae are essentially skin tags that project up from the dentate line or from the junction between the skin and the epithelial lining of the anus (17). The enlargement of existing anal papillae is a consequence of a chronic inflammatory process and fibrotic proliferation within the range of the dentate line, the anorectal zone, and the distal rectal mucosa (18). Most patients remain asymptomatic, but as the anal papillae enlarge to a certain extent, patients may experience itching, wetness, incomplete evacuation, bleeding, or pain (19). Hypertrophic anal papillae often coexist with internal hemorrhoids accompanied by prolapse or bleeding, which can exacerbate patients’ discomfort. The removal of hypertrophic anal papillae. This approach not only eliminates discomfort such as protrusion, bleeding, and anal itchiness caused by anal papillae hypertrophy and internal hemorrhoids but also prevents the risk of malignant transformation due to repeated inflammatory of hypertrophic anal papillae.

The most commonly used surgical procedures for treating Grade IV hemorrhoids are the MMH and the Procedure for Prolapse and Hemorrhoids (PPH). MMH is an open surgery that involves the excision of hemorrhoidal cushions, mucosal tissues, and anal canal skin, offering a strong curative effect and thereby ensuring a low recurrence rate (7, 13, 14). It is currently considered the safest and most effective treatment option. However, due to the significant trauma involved, postoperative pain is generally 3–5 times more severe than with PPH (14, 20). Hospital stays typically last 5–7 days and returning to normal work and daily activities requires 2–3 weeks. Risks of bleeding, infection, and anal stenosis are relatively high. Conversely, the newer PPH procedure involves the excision of only a circumferential mucosal ring, avoiding skin removal, which results in less bleeding and mild postoperative pain (20, 21). Patients usually have a hospital stay of 1–2 days and resume normal activities within 3–7 days. However, its long-term efficacy for fourth-degree hemorrhoids is unstable, with a significantly higher recurrence or prolapse rate compared to MMH (13, 20, 21). PPH is more suited for Grade III hemorrhoids (those that can be repositioned) or as an adjunct prior to MM to reduce hemorrhoidal mass size.

The endoscopic method has not been compared to with these traditional procedures through comprehensive studies. More extensive sample sizes are needed in the future to evaluate its safety and efficacy really. However, our technique offers potential advantages over the established procedures for Grade IV hemorrhoids, including visualized operation and synergy potential with colonoscopy. Modern high-definition endoscopes offer magnification and a seamless view of the surgical field, allowing for clear visualization of the dentate line, anal columns, anal sinuses, and the morphology, color, surface vascular patterns of hemorrhoids, as well as the precise location and size of anal papillae. It helps doctors to more properly find and excise hemorrhoids, reducing tissue damage and lowering the risk of difficulties. The patient experienced minimal bleeding during surgery, slight postoperative pain, was able to ambulate and move freely soon after, and recovered quickly. Moreover, no recurrence of symptoms such as prolapse or defecation difficulties was reported at the one-month and six-month follow-up. Endoscopic follow-up at 8 months showed: No obvious scarring or stricture at the anal canal, the operative site has healed well.

Another advantage of endoscopic treatment for hemorrhoids is the ability to be used in conjunction with colonoscopy. This allows for the simultaneous identification and treatment of other colorectal conditions, such as colon polyps and colorectal cancer, during hemorrhoids surgery. In this case, a colon polyp was removed endoscopically, and hemorrhoids were removed electrocauterically using a snare simultaneously. It reduced the overall recuperation period, avoided the discomfort of several surgeries, saved time and money on medical bills, and enhanced the overall result of treatment. Additionally, patients just require a single intravenous anesthetic, which lowers the risk and pain connected to surgical epidural anesthesia or multiple anesthesia.

While this study provides insights into the application of a new technique for the treatment of Grade IV hemorrhoids, there are also limitations to consider. Firstly, this is a single-case study without a control group, which limits the ability to broadly infer the effectiveness and safety of the technique. Secondly, with a follow-up period of only 8 months, it is relatively short for assessing long-term recurrence rates for Grade IV hemorrhoids. Additionally, individual clinical characteristics of the patient may have significantly influenced the results, which means the success of this technique in this case cannot be assumed to be applicable to a wider patient population. Therefore, despite the potential advantages shown by the current results, larger-scale controlled studies with longer follow-up periods are necessary to fully assess the generalizability and durability of this technique.

## 4 Conclusion

Endoscopic anal papillectomy combined with ligation, leveraging its exceptional visualization capabilities and natural integration with multimodal examination, offers a promising minimally invasive, precise, and function-preserving alternative for treating complex Grade IV prolapsed hemorrhoids. However, its definitive status in clinical guidelines awaits solid evidence from large-scale, prospective, randomized controlled trials assessing its long-term efficacy, particularly in terms of recurrence rates and functional outcomes. At this stage, it is more suitable to be performed by experienced colorectal endoscopists under strict case selection, serving as an important supplement and advanced option beyond traditional surgical methods.

## Data Availability

The original contributions presented in the study are included in the article/**Supplementary Material**. Further inquiries can be directed to the corresponding authors.
